# Ancestry and frequency of genetic variants in the general population are confounders in the characterization of germline variants linked to cancer

**DOI:** 10.1186/s12881-020-01033-x

**Published:** 2020-05-06

**Authors:** Anna Bobyn, Mehdi Zarrei, Yuankun Zhu, Mary Hoffman, Darren Brenner, Adam C. Resnick, Stephen W. Scherer, Marco Gallo

**Affiliations:** 1 Charbonneau Cancer Institute, Cumming School of Medicine, University of Calgary, Calgary, Alberta Canada; 2 Alberta Children’s Hospital Research Institute, Cumming School of Medicine, University of Calgary, Calgary, Alberta Canada; 3 Department of Biological Sciences, Faculty of Science, University of Calgary, Calgary, Alberta Canada; 4 The Centre for Applied Genomics and Program in Genetics and Genome Biology, The Hospital for Sick Children Research Institute, Toronto, Ontario Canada; 5 Department of Molecular Genetics, University of Toronto, Toronto, Ontario Canada; 6 McLaughlin Centre, University of Toronto, Toronto, Ontario Canada; 7 The Children’s Hospital of Philadelphia, Philadelphia, PA USA; 8 Department of Biochemistry and Molecular Biology, Cumming School of Medicine, University of Calgary, Calgary, Alberta Canada; 9 Departments of Oncology and Community Health Sciences, Cumming School of Medicine, University of Calgary, Calgary, Alberta Canada

**Keywords:** Whole-genome sequencing, Single-cell RNA-seq, Pediatric high-grade glioma, Germline variants

## Abstract

**Background:**

Pediatric high-grade gliomas (pHGGs) are incurable malignant brain cancers. Clear somatic genetic drivers are difficult to identify in the majority of cases. We hypothesized that this may be due to the existence of germline variants that influence tumor etiology and/or progression and are filtered out using traditional pipelines for somatic mutation calling.

**Methods:**

In this study, we analyzed whole-genome sequencing (WGS) datasets of matched germlines and tumor tissues to identify recurrent germline variants in pHGG patients.

**Results:**

We identified two structural variants that were highly recurrent in a discovery cohort of 8 pHGG patients. One was a ~ 40 kb deletion immediately upstream of the *NEGR1* locus and predicted to remove the promoter region of this gene. This copy number variant (CNV) was present in all patients in our discovery cohort (*n* = 8) and in 86.3% of patients in our validation cohort (*n* = 73 cases). We also identified a second recurrent deletion 55.7 kb in size affecting the *BTNL3* and *BTNL8* loci. This *BTNL3–8* deletion was observed in 62.5% patients in our discovery cohort, and in 17.8% of the patients in the validation cohort. Our single-cell RNA sequencing (scRNA-seq) data showed that both deletions result in disruption of transcription of the affected genes. However, analysis of genomic information from multiple non-cancer cohorts showed that both the *NEGR1* promoter deletion and the *BTNL3–8* deletion were CNVs occurring at high frequencies in the general population. Intriguingly, the upstream *NEGR1* CNV deletion was homozygous in ~ 40% of individuals in the non-cancer population. This finding was immediately relevant because the affected genes have important physiological functions, and our analyses showed that *NEGR1* expression levels have prognostic value for pHGG patient survival. We also found that these deletions occurred at different frequencies among different ethnic groups.

**Conclusions:**

Our study highlights the need to integrate cancer genomic analyses and genomic data from large control populations. Failure to do so may lead to spurious association of genes with cancer etiology. Importantly, our results showcase the need for careful evaluation of differences in the frequency of genetic variants among different ethnic groups.

## Background

Brain cancers have recently surpassed leukemias as the number one killer in the pediatric cancer patient population [1]. This appears largely attributable to significant improvements in the clinical management of some leukemia subtypes, whereas no significant progress has been registered for the malignant brain cancer population.

Pediatric high-grade gliomas (pHGGs; World Health Organization grade III and IV tumors), including glioblastoma (GBM), have particularly dismal prognoses [2]. Current treatments usually include maximal safe resection of the main tumor mass followed by local radiotherapy. Some pHGG patients also receive chemotherapy, although this treatment is not uniform and varies depending on the specific patient, prescribing oncologist and treating centre. Temozolomide, which has shown some efficacy in prolonging overall survival in adult GBM patients, is sometimes administered to pHGG patients as well, although clinical trials failed to show efficacy for this drug in pediatric cohorts [3, 4]. New treatment options are therefore needed to tackle these universally lethal malignancies.

Several genomic studies have shown that pHGGs have low mutational burdens, similarly to other childhood cancers [5–8]. The mutational landscape of pHGGs is very different from their adult counterparts. For instance, our analyses using cBioPortal and pedcBioPortal show that whereas *EGFR* is mutated in 53% of adult GBM samples, and *PTEN* is altered in 31% of cases (*n* = 281 samples described in reference [9]), these genes are mutated in 6 and 4% of pHGG cases, respectively (*n* = 1257 cases described in references [10, 11]). Highly recurrent mutations in pHGGs include mutations of genes encoding the histone 3 variant H3.3, including 21% of cases with mutations in the *H3F3A* gene. H3.3 mutations tend to co-occur with *TP53* and *ATRX* mutations, and are very rare in adult HGGs [6, 12, 13].

Molecular studies and work with genetic mouse models have shown that co-occurrence of H3.3 and *Tp53* mutations cooperate with either overexpression of *Pdgfra* or loss of *Nf1* to drive cancer initiation and progression [14, 15]. However, the majority of human pHGG cases lack these concurrent mutations and their genetic drivers are difficult to infer.

We have recently reported a whole-genome sequencing (WGS) analysis of a collection of pHGGs [5]. In that study, we showed that pHGGs are genomically complex cancers that harbor multiple coexisting genetic subclones. Among the truncal mutations (ie variants that are shared by virtually all the subclones detected in a tumor), we found no obvious candidate driver events in most tumors, except for the above-mentioned H3.3/*TP53*/*ATRX* axis.

Traditionally, somatic mutations are called by comparing WGS data for the tumor tissue and germline (usually peripheral blood) to subtract variants that are specific to the individual patient. The underlying assumption of this method is that germline variants are not informative for cancer etiology. However, recent publications have shown that about 7–8% of pediatric cancer patients harbor deleterious mutations in at least one of 149 genes with known association to cancer [16]. This frequency might be an underestimation because more than these 149 genes might drive specific cancer types. We hypothesized that the lack of clear genetic drivers in the majority of pHGGs might be an artifact due to the removal of informative germline events that could predispose an individual to the development of the malignancy and/or affect disease progression. Therefore, we analyzed germline and tumor WGS data separately, and then looked specifically for structural variants that were shared between the germline and the tumor tissue and that recurred in multiple pHGG patients. Our analyses identified two structural variants that were highly recurrent in the pHGG population. However, subsequent analyses with datasets derived from a control population of thousands of individuals revealed that these variants are present at high frequency in the non-cancer population. Of interest, we found that these variants occurred with different frequencies in different ethnic groups.

Our findings highlight the need to contextualize findings from cancer genomic studies with genomics data from non-cancer cohorts in order to properly identify putative cancer predisposing genes. This is especially relevant now that significant efforts are being invested in uncovering predisposing germline variants for different cancer types, including adult malignancies. This task will be increasingly enabled by efforts from large consortia that are collecting genomic information from the general population.

## Methods

### pHGG samples

Samples used for WGS with linked reads for the pHGG discovery cohort (*n* = 8 patients) were recently described in Hoffman et al. [5].

### Non-cancer control cohort

The large non-cancer control cohort comprises of 2596 genome sequences hosted at the Centre for Applied Genomics at the Hospital for Sick Children, Toronto [17]. They are parents and unaffected siblings of individuals from our disease sequencing studies. We also analyzed other population control data from Personal Genome Project Canada (PGPC; *n* = 93) [18] and 1000 Genomes Project CNVs obtained from the Database of Genomic Variants (DGV; *n* = 2504) [19].

### Visualization of genomic data

Data generated by WGS with linked reads were visualized with Loupe version 2.1.2 (10xGenomics). scRNA-seq data were visualized with Loupe Cell Browser version 3.0.1 (10xGenomics). The single-cell transcriptomics data for human hippocampus and cortex [20] was accessed and analyzed through the Single Cell Portal (https://singlecell.broadinstitute.org/single_cell), a web interface hosted by the Broad Institute.

### Survival plots

Survival analysis was done using a previously published pHGG cohort [21] with the R2 Genomic Analysis Visualization Platform (https://hgserver1.amc.nl/cgi-bin/r2/main.cgi). Patients were stratified based on *NEGR1* expression, with *NEGR1*-low cases corresponding to the bottom quartile of expression. Statistical analysis was performed with the log-rank test.

### Graphing software

Pie charts and histograms were generated with Prism 8 (GraphPad).

## Results

### Identification of recurrent genetic variants at the *NEGR1* locus in pHGGs

**Fig. 1:**
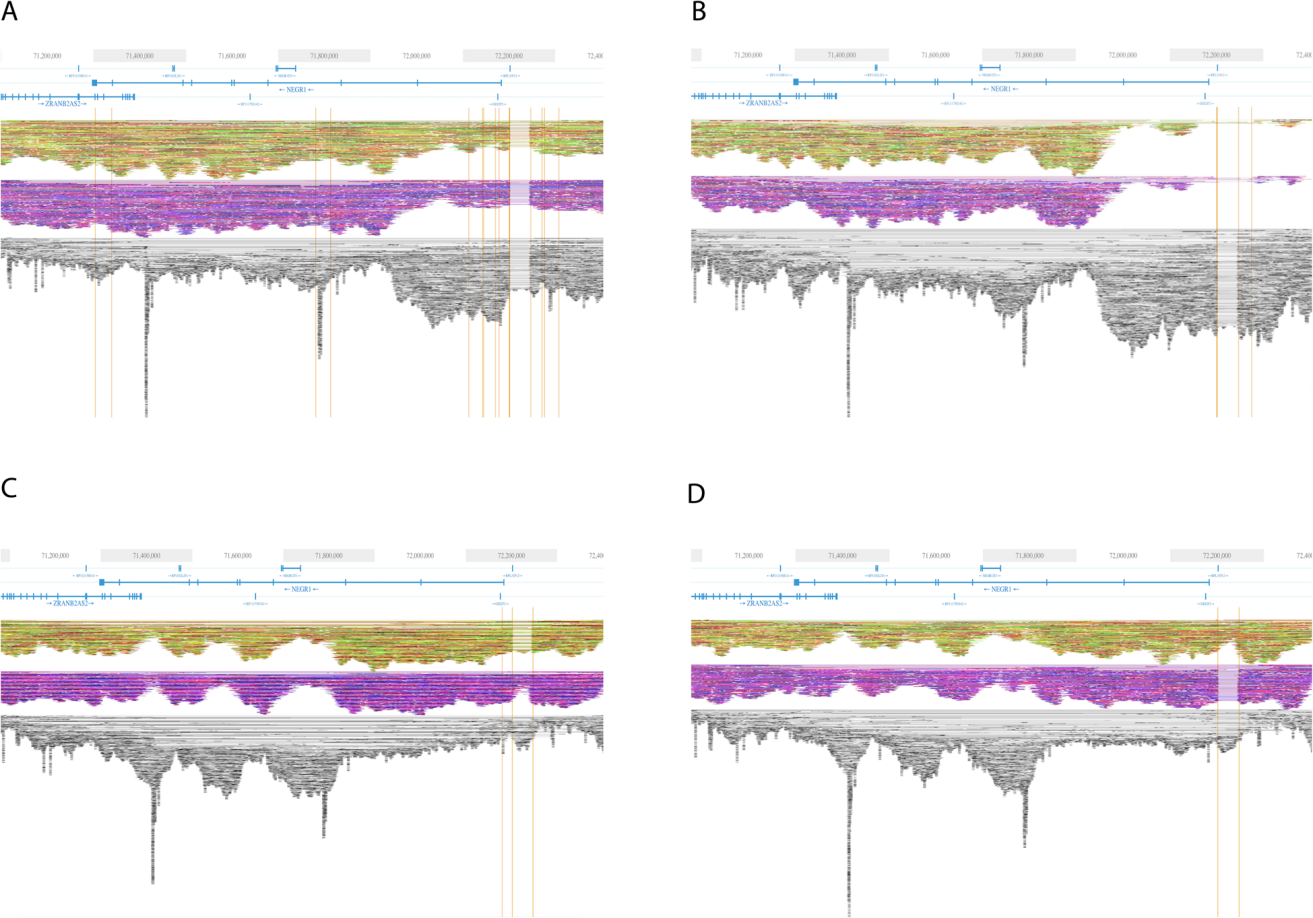
Linked-read sequencing data for two pHGG patients at the *NEGR1* locus. **a**. Homozygous *NEGR1* deletion in the tumor profile of patient 6 (G641). **b**. Homozygous deletion in the germline of patient 6 (G641B). **c**. Heterozygous *NEGR1* deletion in the tumor profile of patient 1 (SM2932). **d**. Heterozygous deletion in the germline of patient 1 (SM2819). In all panels, linked-reads are organized in haplotype blocks. Each haplotype is color-coded (green/yellow or pink/purple). Unassigned linked-reads are shown in back/gray at the bottom of each panel

We have profiled pHGG samples by WGS with linked-read technology (10xGenomics), as recently described [5]. Linked-reads allow the reconstruction of long (Mbp) haplotypes at the level of individual chromosomes and are optimal for the identification and visualization of structural variants. In particular, because maternal and paternal haplotypes are determined by analysis of single-nucleotide polymorphisms along the chromosome length, this experimental set up allows assignment of structural variants to a specific haplotype. We generated WGS datasets for matched tumor tissue and blood samples (germline controls) from a discovery cohort of 8 pHGG patients. In addition, relapse samples were available for 4 patients, including 3 relapses for one patient (patient samples and anagraphical information were described in ref. [5] and are summarized in Supplemental Table S1). We were surprised to observe 100% of our tumor samples displaying a deletion immediately upstream of the Neuronal Growth Regulator 1 (*NEGR1*) gene. Five out of eight had homozygous deletions (see Fig. 1a-b for examples), whereas three out of eight pHGG patients from our cohort harboured a heterozygous deletion in the *NEGR1* region (Fig. 1c-d; Table 1). The deletions were found both in the germline and in tumor tissue. The NEGR1 protein is a member of the IgLON subgroup of the immunoglobin superfamily and has been shown to contain a GPI-anchor attachment site that localizes to lipid rafts [22] and is involved in the maturation and remodelling of the central nervous system [23]. Knockout of the *Negr1* gene in mouse models results in defective neuronal maturation [24]. The cell adhesion molecule encoded by NEGR1 has also been reported to be down-regulated in many human cancers. In ovarian cancer, *NEGR1* has been proposed as a tumour suppressor gene [22]. Additionally, low NEGR1 expression is correlated with a low survival probability in neuroblastoma, according to a previous study [25]. Given the role of *NEGR1* in neural development and cancer progression, this gene proves to be an intriguing subject in brain cancer research. Additionally, our data raised the possibility that mutations in this gene may be germline-predisposing events and deserved follow up studies.

**Table 1:** Summary of the frequencies of *NEGR1* and *BTNL8-BTNL3* deletion

	NEGR1 deletion	BTNL8-BTNL3 deletion
Calgary Cohort (n = 8)	100%	62.5%
CBTTC (n = 73)	86.3%	17.8%
MSSNG controls (*n* = 2596)	87.1%	48.0%
1000 Genomes Project (*n* = 2504)	89%	38.2%
Personal Genome Project Canada (n = 93)	77.4%	48.4%

Datasets include the Calgary cohort, a pediatric HGG dataset from the CBTTC and individuals from the general population (coded parental control Canadian samples in MSSNG); 1000 Genomes Project CNVs obtained from the Database of Genomic Variants [DGV]; and control samples from Personal Genome Project Canada (PGPC)). Deletions are either heterozygous or homozygous

Datasets include the Calgary cohort, a pediatric HGG dataset from the CBTTC and individuals from the general population (coded parental control Canadian samples in MSSNG); 1000 Genomes Project CNVs obtained from the Database of Genomic Variants [DGV]; and control samples from Personal Genome Project Canada (PGPC)). Deletions are either heterozygous or homozygous.

### Low *NEGR1* expression is associated with worse prognosis in pHGGs

Because the deletion we observed at the *NEGR1* locus was immediately upstream of the gene, we predicted that this lesion might affect the ability of the promoter region to properly activate transcription. Analysis of ENCODE data for histone marks linked to active promoter and enhancer elements supported the notion that the deletion might remove regions that are important for *NEGR1* transcription (Supplemental Figure S1).

**Fig. 2:**
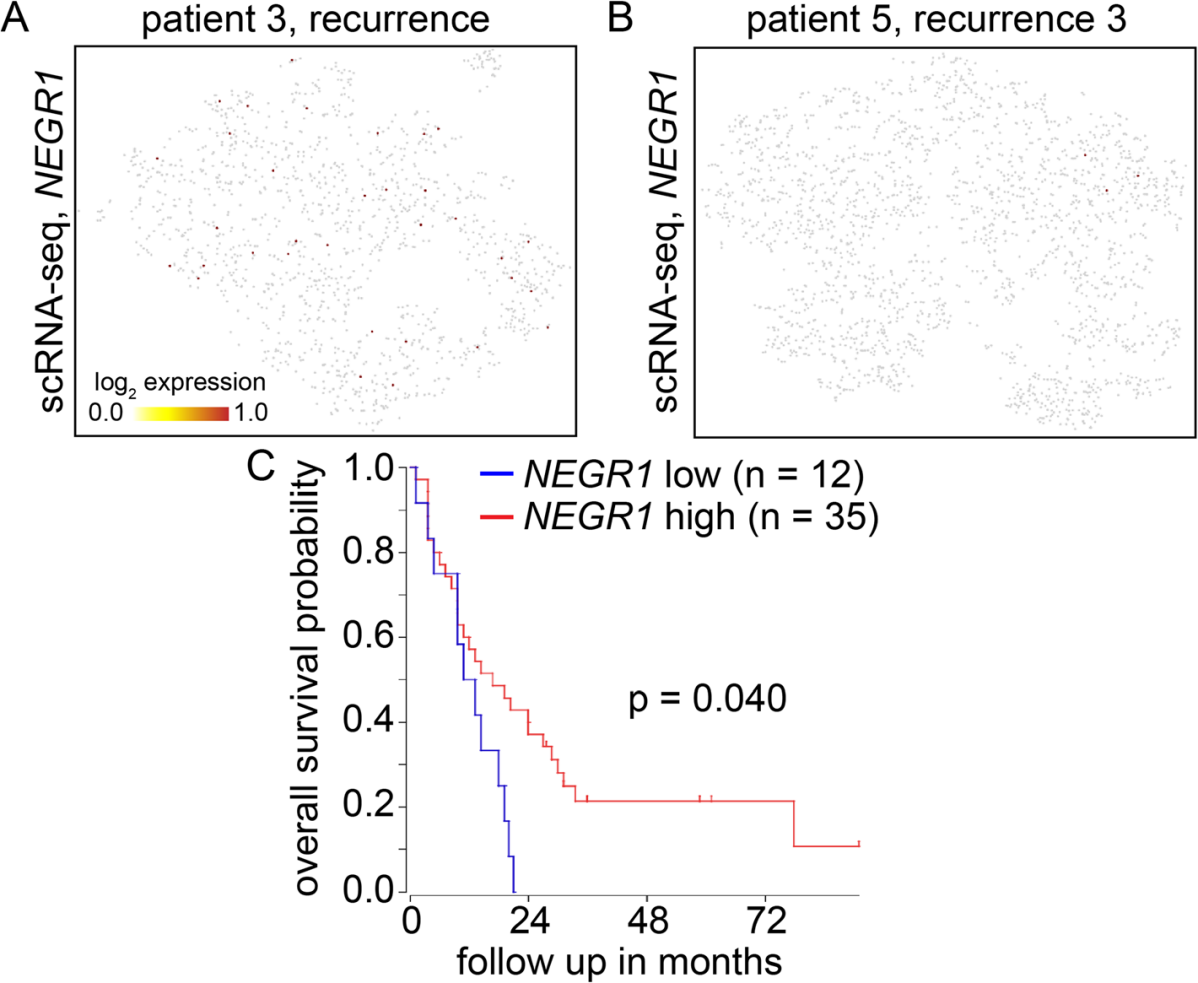
Single cell RNA-sequencing of *NEGR1* expression levels. **a**. tSNE plot showing single cell RNA-sequencing data illustrates *NEGR1* transcription levels in a xenograft derived from recurrence one of patient 3. **b**. tSNE plot showing *NEGR1* transcription levels in single cell RNA-sequencing datasets generated from a xenograft derived from the third recurrence of patient 5. **c**. A Kaplan-Meier Curve for patient populations with either high or low expression of *NEGR1*

To further assess the possibility that the deletion upstream of *NEGR1* might affect the expression of this gene in pHGG, we re-analyzed single-cell RNA-seq data that our group recently generated from two patient-derived xenografts [5] and looked specifically at expression of *NEGR1* in these samples. Both xenografts were derived from samples in the Calgary cohort that were profiled with linked-read WGS and had homozygous deletions upstream of *NEGR1*. We found that neither xenograft expressed appreciable amounts of *NEGR1* (Fig. 2a,b). In contrast, transcription of *ZRANB2*, a gene immediately downstream of *NEGR1*, was detected in our scRNA-seq datasets (Supplemental Figure S2A,B). In addition, transcription of *NEGR1* was detected in previously published single-cell transcriptomic datasets generated from the adult human brain [20] (Supplemental Figure S2C,D). Overall, these data indicate that deletions of the *NEGR1* promoter region in pHGGs may result in abrogation of gene expression in pHGG. However, the role of other factors (including epigenetic mechanisms) in repressing *NEGR1* transcription cannot be ruled out at this time.

We also looked at the effects of *NEGR1* expression on overall survival in a previously published pHGG patient cohort [21]. We found that low expression of *NEGR1* was significantly associated with shorter overall survival in this cohort (Fig. 2c). Overall, our data suggest that genetic events affecting *NEGR1* expression might have deleterious effects on the survival of pHGG patients.

Our discovery cohort was composed of 8 pHGG patients, a number that limits predictions of applicability of our findings to the larger patient population. We therefore explored whether the deletions at the *NEGR1* locus could be identified in a larger cohort of 73 pHGG patients collected by the Children’s Brain Tumour Tissue Consortium (CBTTC) [26]. We found that the deletion upstream of *NEGR1* was present in 63 out of 73 patients (frequency of 86.3%; Table 1).

### Recurrent germline deletions at the *BTNL3* and *BTNL8* loci in pHGG patients

**Fig. 3:**
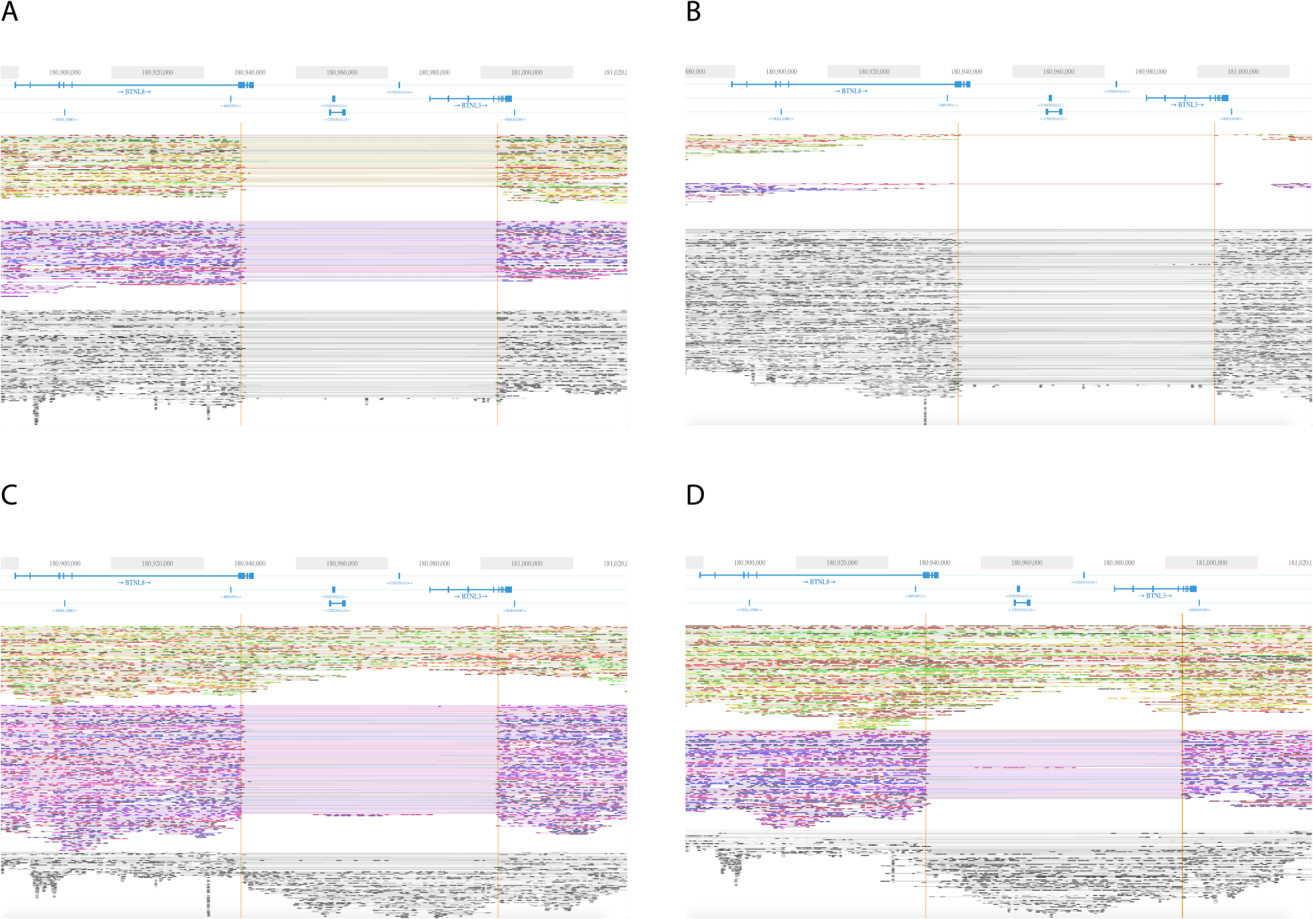
Linked-read sequencing data for two pHGG patients at the *BTNL8-BTNL3* locus. **a** Homozygous *BTNL8-BTNL3* deletion in the tumor profile of patient 6 (G641). **b** Homozygous deletion in the germline of patient 6 (G641B). **c** Heterozygous *BTNL8-BTNL3* deletion in the tumor profile of patient 1 (SM2932). **d** Heterozygous deletion in the germline of patient 1(SM2819)

Intrigued by these findings, we searched for other recurrent germline structural variants in our WGS datasets. We observed frequent deletions (55.7 kb) in the genomic region encompassing the genes *BTNL3* and *BTNL8*. Overall, this deletion was homozygous in 2 of 8 patients (Fig. 3a, b), and heterozygous in three out of eight pHGG patients (Fig. 3c, d) in our cohort (Table 1). This deletion was also present in patients’ germlines (Fig. 3). Butyrophilin (BTN)-like molecules are a part of the B7 family of proteins, which are involved in immune response. The role of the B7 family in regulating the primary immune response against cancer was previously highlighted in clinical trials using monoclonal antibodies against PD-1 and B7-H1 [27, 28]. BTNL8 has two alternatively spliced forms, B7-like and BTN-like. The extracellular domain has been reported to bind the surface of T cells, co-stimulating proliferation and cytokine production [29]. Although there is little known about the functional role of BTNL3, its downregulation was reported in colon cancer alongside BTNL8 [30]. The frequency of the *BTNL3–8* deletion was 17.8% in the pHGG CBTTC cohort (Table 1). These data confirm that this deletion is frequent in the pHGG population.

### Frequency of the *NEGR1* and *BTNL3–8* deletions in the general population

**Fig. 4:**
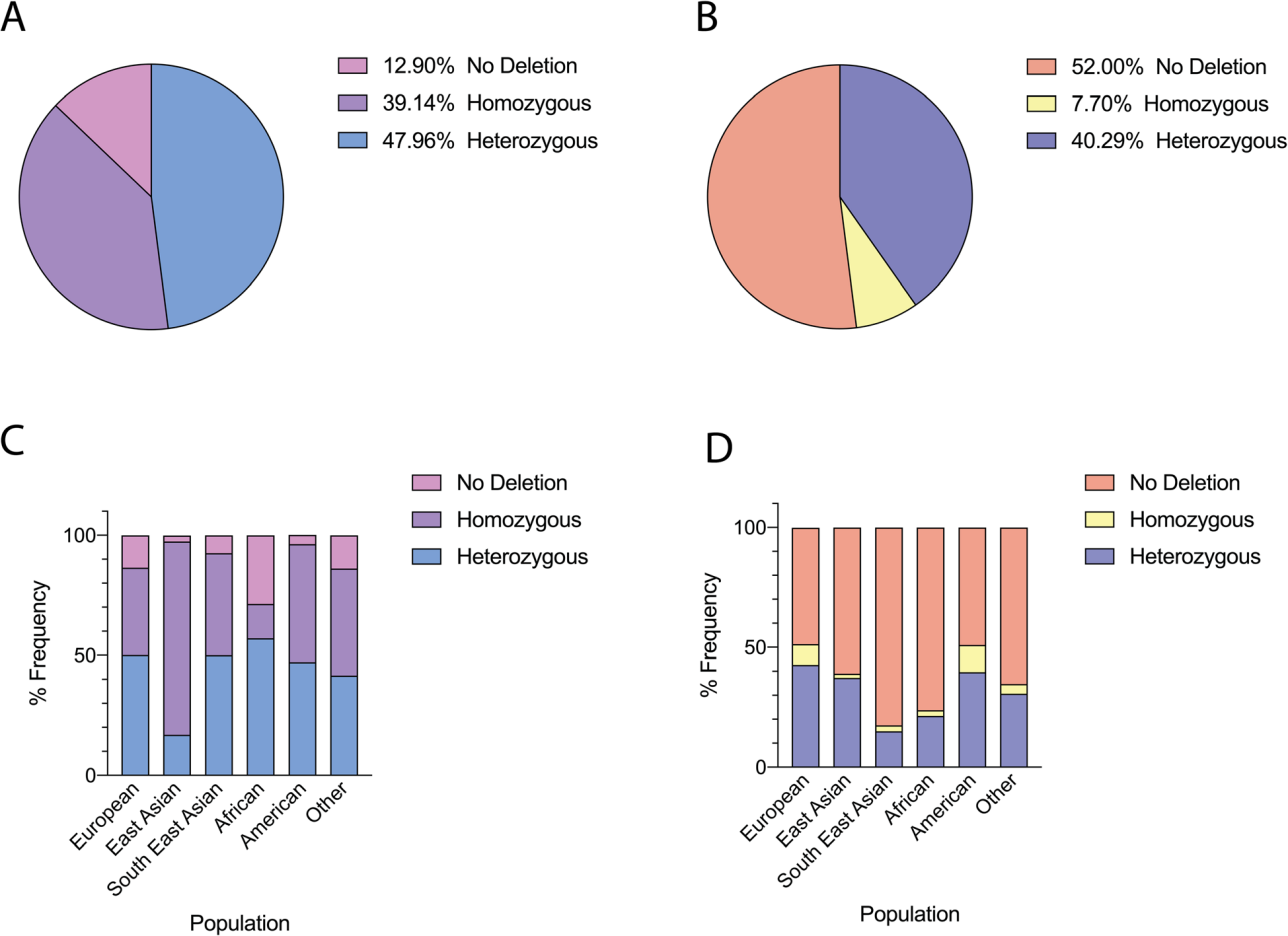
*NEGR1* and *BTNL8-BTNL3* deletion frequencies in the general population. **a**. Frequency of *NEGR1* deletions in the general population for all ethnicities. **b**. Frequency of *BTNL8-BTNL3* deletions in the general population for all ethnicities. **c**. *NEGR1* deletions stratified by European, East Asian, South East Asian, African, American, and “Other” descent. **d**. *BTNL8-BTNL3* deletions stratified by European, East Asian, South East Asian, African, American, and “Other” descent

The sequence-level breakpoints of the deletions upstream *NEGR1* are chr1:72,766,325-72,811,839 (hg19) and were similar among different ethnicities. However, breakpoints of the deletions impacting BTNL8-BTNL3 occurred in repeat regions, thus the exact coordinates were not identifiable due to the complexity of the genomic region. Because the frequency of deletions at the *NEGR1* and *BTNL3–8* loci was relatively high in pHGG patients, we examined whether these genetic variants were specific to or enriched in the pHGG population. We therefore determined the frequency of these deletions in a large non-cancer cohort that includes genomic information on 2596 individuals [17]. We found that this population control dataset had an *NEGR1* deletion frequency of 87.1% (Table 1; Fig. 4a), comparable to the frequency (86.3%) that we observed in the CBTTC pHGG cohort. Our results also show that the *BTNL3–8* deletion was detectable in 48.0% of the controls assessed (Table 1; Fig. 4b), higher than the frequency (17.8%) we observed in the CBTTC pHGG cohort (Table 1). Contrary to our expectations, these data indicate that the *NEGR1* and *BTNL3–8* germline deletions are relatively common in the general population, and do not appear to be specifically over-represented in the pHGG population. We also analyzed 1000Genome WGS datasets (*n* = 2504) with copy number variation (CNV) deposited in the Database of Genomic Variants (DGV) [19, 31], which is the most comprehensive curated public open-source repository for CNVs from population controls. We found a frequency of 89% for the deletion upstream of *NEGR1* and 38.2% for that impacting *BTNL3–8*, an observation similar to the earlier control findings.

### The frequency of *NEGR1* and *BTNL3–8* deletions varies in different ethnic groups

Further investigation of the non-cancer population revealed frequency differences of the deletions at the *NEGR1* and *BTNL3–8* loci between six human populations: European, East Asian, South East Asian, African, American, and “Other”. At the *NEGR1* locus, the most dramatic difference was observed between the East Asian and African cohorts. The East Asian cohort had the highest frequency of *NEGR1* deletions with only 2.5% of the population having no deletion in comparison to 28.6% in the African population (Fig. 4c, Table 2).

**Table 2:** *NEGR1* and *BTNL8-BTNL3* deletions in population controls

	NEGR1 deletion		BTNL8-BTNL3 deletion	
	Homozygous	Heterozygous	Homozygous	Heterozygous
European (*n* = 2101)	36.4%	50.1%	8.6%	42.7%
East Asian (*n* = 118)	80.5%	16.9%	1.7%	37.3%
South East Asian (*n* = 80)	42.5%	50.0%	2.5%	15.0%
African (*n* = 42)	14.3%	57.1%	2.4%	21.4%
American (*n* = 53)	49.1%	47.2%	11.3%	39.6%
Other (*n* = 202)	44.6%	41.6%	4.0%	30.7%

Frequencies of the control collection (n = 2596) stratified by ethnic groups and homozygous or heterozygous deletion types

Frequencies of the control collection (*n* = 2596) stratified by ethnic groups and homozygous or heterozygous deletion types.

Similarly, the East Asian cohort had homozygous and heterozygous deletions of 80.5 and 16.9% respectively, as opposed to 14.3 and 57.1% in the African population (Fig. 4c). These cohorts were statistically significant with *p*-value < 0.00001 by Chi-Square analysis (Table 3).

**Table 3:** Chi-square analysis of *NEGR1* deletions in the general population

	East Asian	African	American	SE Asian	Other
**European**	91.8	12.8	6.03	2.88	6.00
**East Asian**		62.1	18.0	30.3	40.4
**African**			18.6	15.2	14.6
**American**				1.09	4.13
**SE Asian**					2.90

Analysis of the parental controls from the MSSNG cohort. Values are statistically significant to 95% above the critical value 5.99 (df = 2) and shown in blue. Statistically insignificant results are shown in red

The *BTNL3–8* deletion also had different frequencies between ethnic groups (Fig. 4d, Table 2). The largest differences were observed between European and South East Asian descent, with 48.6 and 82.5% of the respective populations showing no deletion at this locus (Fig. 4d). The European and South East Asian groups were statistically significant with p-value < 0.00001 by Chi-Square analysis (Table 4). These data therefore illustrate the large variability in frequency of germline genetic variants between ethnic groups, a factor that should be incorporated into studies aimed to identify novel germline variants in cancer populations.

**Table 4:** Chi-square analysis of *BTNL8-BTNL3* deletions in the general population

	East Asian	African	American	SE Asian	Other
**European**	10.7	12.7	0.556	35.4	21.5
**East Asian**		3.53	8.21	11.7	2.42
**African**			7.82	0.799	1.87
**American**				17.1	6.97
**SE Asian**					8.12

Analysis of the parental controls from the MSSNG cohort. Values are statistically significant to 95% above the critical value 5.99 (df = 2) and shown in blue. Statistically insignificant results are shown in red

## Discussion

The identification of germline genetic variants that might predispose to cancer is an emerging theme in the field of cancer genomics. The identification of such variants holds the promise to incorporate genetic tests as part of early detection strategies for some cancers. Such strategies would be particularly important for pHGG, which is universally lethal. High-profile studies have shown that a significant fraction of the pediatric cancer population carries germline variants in genes known to be cancer drivers or that are associated with cancer etiology and progression [16]. We think it is important to stress that most of the evidence to define these variants as “drivers” derived from studies of adult cancers. It is however possible that the mutational dependencies of childhood and adult cancers might be divergent. This case is well exemplified by the radically different incidence of specific genetic alterations in *EGFR* and *H3F3A* in pediatric and adult HGGs, as we mentioned in the introduction to this manuscript. There is therefore promise in efforts to identify new genetic variants that may act as specific drivers of childhood cancers.

Here, we highlight potential confounding factors in the process of identification of new candidate germline variants associated with cancer. Specifically, our work identified two variants affecting genes that were very attractive candidate cancer-predisposing loci, based on their known function and previously published evidence of their involvement in several malignancies. However, these variants were relatively frequent in non-cancer human populations, with marked differences in frequency based on ancestry.

We have identified highly recurrent deletions at two sites - *NEGR1* and *BTNL3–8* - in the genomes of pHGG patients. From the perspective of a discovery platform, both sites were intriguing because of the biological functions of the genes affected by the lesions. *NEGR1* was previously shown to have an important role in neural development [23, 24]. In particular, work with genetic mouse models showed that *Negr1* is required for terminal differentiation of neurons and for their ability to properly form synapses. The deletions we identified in pHGG patients are predicted to affect the regulatory regions of the gene. This prediction is supported by our scRNA-seq data, which showed undetectable levels of *NEGR1* transcripts in two patient-derived xenograft models. Based on all these data, it would be reasonable to conclude that *NEGR1* may play a role in the etiology of pHGG.

However, our analyses of non-cancer populations clearly show that the *NEGR1* promoter deletion is present in a majority of individuals in the general population. Based on this finding, it is therefore difficult to support the notion that *NEGR1* might be involved in tumor etiology in the context of pHGG, and possibly other cancers as well. We found, however, an association between low expression of *NEGR1* and poor overall survival in pHGG patients. It is therefore possible that deletions that negatively affect *NEGR1* expression might have modulatory effects on brain tumors and have negative prognostic value. This would be interesting, because it would exemplify that some common germline variants could have effects on tumor progression.

Our finding that the region upstream of *NEGR1* is homozygously deleted in ~ 40% of individuals in the general population is particularly intriguing. Since mouse models with homozygous *Negr1* deletions have neural defects, our data raise the question of whether the murine and human orthologues paly similar roles in brain development. Our data seem to challenge this notion. Another possibility is that the human lineage developed compensatory mechanisms that can overcome loss of *NEGR1* expression during neural development, whereas *Negr1* plays a more pivotal role during mouse development.

Recent publications have shown that some cancer patients carry deleterious variants of established cancer genes in their germlines, suggesting that some individuals may be predisposed to developing some malignancies [16, 32]. Cancer initiation and progression may therefore be modulated by the interplay and crosstalk between germline and somatic variants. Our present work highlights the need for comparing the frequencies of putative cancer predisposition variants in the germlines of cancer patients and non-cancer populations. A cancer-centric perspective may result in the identification of germline variants that are relatively frequent in the general population. These comparisons are made easier by large genomic datasets that are being collected by international efforts.

**Fig. 5:**
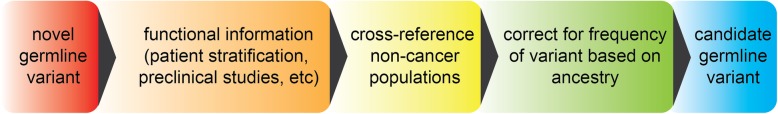
Model workflow for the identification of novel candidate germline variants associated with cancer. We suggest several filters to identify candidate cancer germline variants. As a first step, information on whether the variant itself or the transcription levels of its associated gene can stratify patients based on survival should be considered. Next steps should include comparing variant frequency in cancer and non-cancer populations, and adjusting for the ancestry of the cancer and non-cancer cohorts. These steps could streamline the identification of candidate germline variants associated with a specific cancer type, and which should be selected for further validation

In addition, our data show major differences in the frequencies of the deletions at the *NEGR1* and *BTNL3–8* loci between different ethnic groups. These results highlight the need to cross-reference the frequencies of germline variants with non-cancer populations with appropriate ethnic backgrounds (Fig. 5). The magnitude of this problem was recently highlighted in a review article, which reported that 78% of people recruited in genomic studies is of European ancestry [33]. These are traditional concepts in the field of genetic association studies that will have to be incorporated more thoroughly into cancer genomic studies. This need is made even more urgent because of the recent emphasis on research that aims to identify germline predisposing events in cancer patients.

## Conclusions

We found high-frequency deletions upstream of the *NEGR1* locus in pHGG and non-cancer cohorts. Low *NEGR1* expression may be correlated with worse prognosis for pHGG patients. Our data underscore the need for efforts to identify new cancer-predisposing germline genetic events to use control populations that have been appropriately stratified based on ancestry.

## Supplementary information

**Additional file 1 : Supplemental Figure S1.** UCSC Genome Browser view of the *NEGR1* locus. Layered ChIP-seq tracks for the active enhancer histone mark H3K27Ac, DNase clusters (corresponding to accessible chromatin) and transcription (Txn) factor binding data from the ENCODE project are shown. The data indicate that the deleted region upstream of *NEGR1* in pHGG patients may harbor regulatory regions. **Supplemental Figure S2.** The deletion upstream of *NEGR1* appears to have specific effects on the transcription of this gene. (A-B) Transcription of *ZRANB2*, the gene immediately downstream of *NEGR1*, is detected in our scRNA-seq datasets. (C) Single-cell transcriptomics data for 14,963 cells isolated from human hippocampus and cortex. This tSNE plot describes the clustering of different cell populations present in these brain regions. (D) Transcription of *NEGR1* is detected in single-cells isolated from human hippocampus and cortex. **Supplemental Table S1.** Patient and sample information.

## Data Availability

WGS datasets were described in Hoffman et al. [5] and were deposited to the European Genome-phenome Archive (EGA) under accession number EGAS00001003432 (https://ega-archive.org/studies/EGAS00001003432). scRNA-seq datasets were also described in Hoffman et al. [5] and were deposited to the Gene Expression Omnibus (GEO) under accession number GSE117599. The CBTTC dataset is hosted on Kids First Data Resource Portal and can be accessed via DOI:10.24370/SD_BHJXBDQK. Population genetic analysis of CNVs used publicly available data in the Database of Genomic Variants (DGV) via http://dgv.tcag.ca/dgv/app/home. Initial assessment of the CNVs tested Canadian parental controls present in the MSSNG dataset, which is an open science resource available through a Data Access Committee (see https://www.mss.ng). PGCP genome data files are publicly available at www.personalgenomes.ca

## Abbreviations

CBTTC: Children’s Brain Tumor Consortium
CNV: Copy number variation
DGV: Database of Genomic Variants
GBM: Glioblastoma
pHGG: Pediatric high-grade glioma
scRNA-seq: Single-cell RNA sequencing
WGS: Whole-genome sequencing
