# Supplementary material for: Ancestry and frequency of genetic variants in the general population are confounders in the characterization of germline variants linked to cancer
Source: BMC Med Genet. 2020 May 6;21:92. doi: 10.1186/s12881-020-01033-x (PMC7201963; doi:10.1186/s12881-020-01033-x)
Supplement: Supplementary file 1 — Additional file 1 : Supplemental Figure S1. UCSC Genome Browser view of the NEGR1 locus. Layered ChIP-seq tracks for the active enhancer histone mark H3K27Ac, DNase clusters (corresponding to accessible chromatin) and transcription (Txn) factor binding data from the ENCODE project are shown. The data indicate that the deleted region upstream of NEGR1 in pHGG patients may harbor regulatory regions. Supplemental Figure S2. The deletion upstream of NEGR1 appears to have specific effects on the transcription of this gene. (A-B) Transcription of ZRANB2, the gene immediately downstream of NEGR1, is detected in our scRNA-seq datasets. (C) Single-cell transcriptomics data for 14,963 cells isolated from human hippocampus and cortex. This tSNE plot describes the clustering of different cell populations present in these brain regions. (D) Transcription of NEGR1 is detected in single-cells isolated from human hippocampus and cortex. Supplemental Table S1. Patient and sample information. [file 12881_2020_1033_MOESM1_ESM.pdf]

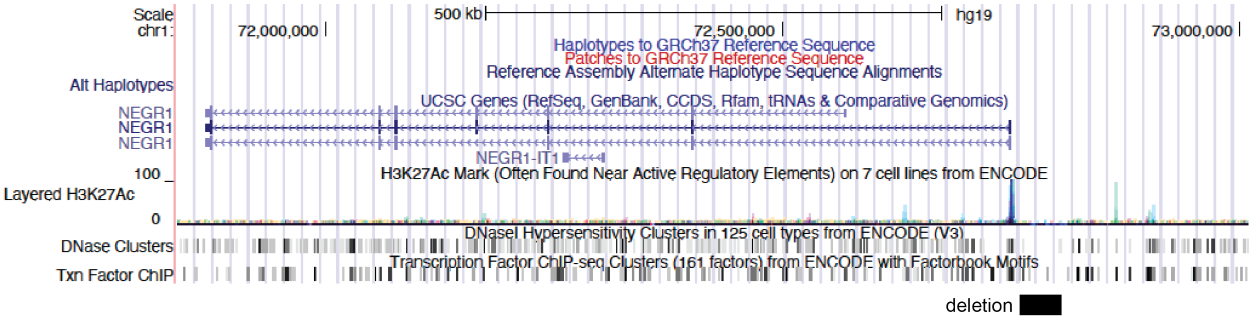

Supplemental Figure S1. UCSC Genome Browser view of the *NEGR1* locus.
Layered ChIP-seq tracks for the active enhancer histone mark H3K27Ac, DNase clusters (corresponding to accessible chromatin) and transcription (Txn) factor binding data from the ENCODE project are shown. The data indicate that the deleted region upstream of *NEGR1* in pHGG patients may harbor regulatory regions.

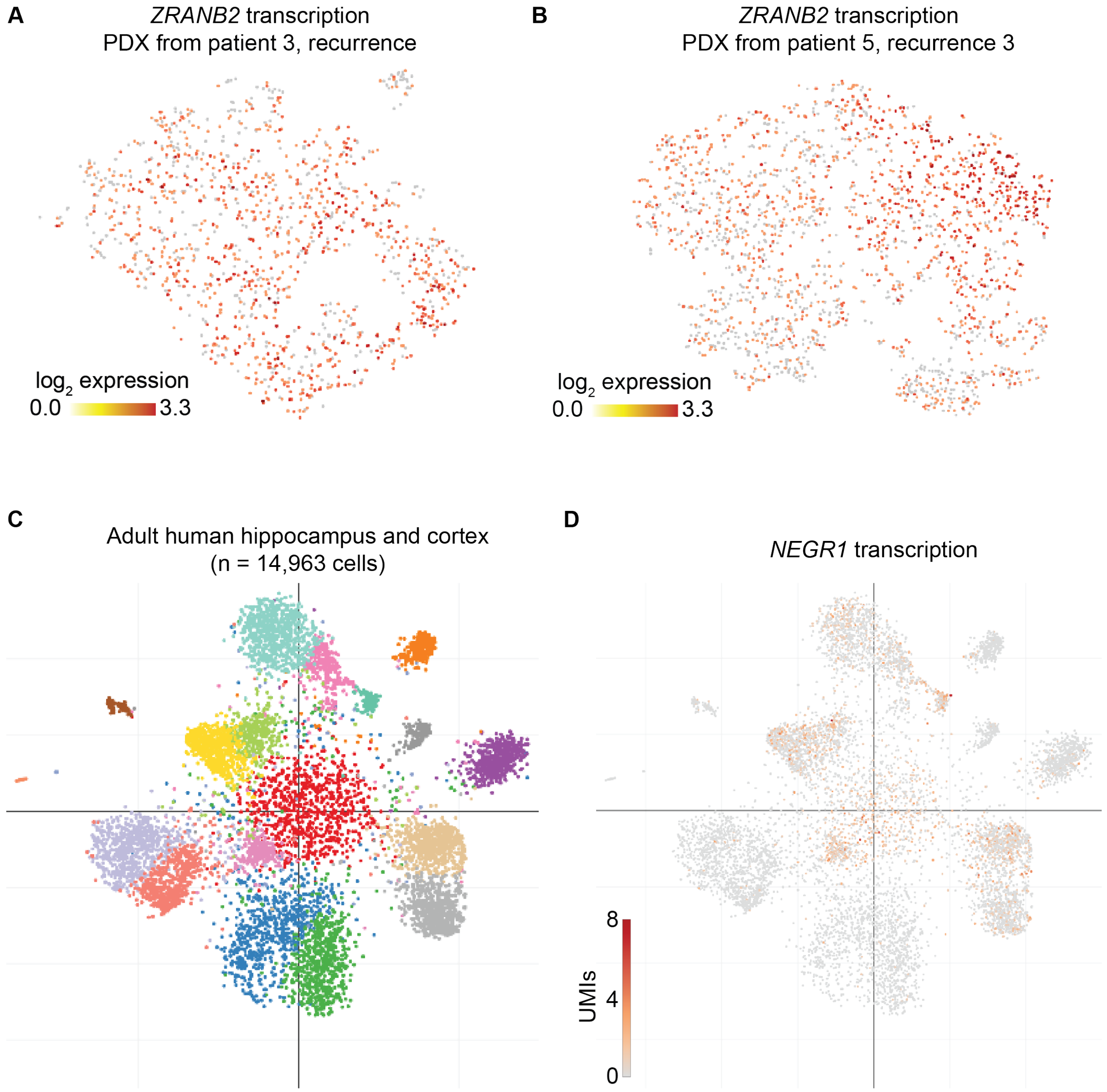

Supplemental Figure S2. The deletion upstream of *NEGR1* appears to have specific effects on the transcription of this gene.
(A-B) Transcription of *ZRANB2*, the gene immediately downstream of *NEGR1*, is detected in our scRNA-seq datasets.
(C) Single-cell transcriptomics data for 14,963 cells isolated from human hippocampus and cortex. This tSNE plot describes the clustering of different cell populations present in these brain regions.
(D) Transcription of *NEGR1* is detected in single-cells isolated from human hippocampus and cortex.

patient #	diagnostic sample	relapse 1	relapse 2	relapse 3	blood (germline)
1	SM2932	SM2937			SM2819
2	SM3013	SM3042			SM2907
3	SM3749	SM4021			SM3375
4	SM3759				SM3787
5	SM4018	SM4025	SM4058	SM4063	SM3623
6	G641				G641B
7	G697				G697B
8	G578				G578B

Supplemental Table S1. Patient and sample information.
